# Vitamin D Status and Potential Therapeutic Options in Critically Ill Patients: A Narrative Review of the Clinical Evidence

**DOI:** 10.3390/diagnostics12112719

**Published:** 2022-11-07

**Authors:** Salvatore L. Cutuli, Laura Cascarano, Eloisa S. Tanzarella, Gianmarco Lombardi, Simone Carelli, Gabriele Pintaudi, Domenico L. Grieco, Gennaro De Pascale, Massimo Antonelli

**Affiliations:** 1 Department of Anesthesiology, Intensive Care and Emergency Medicine, Fondazione Policlinico Universitario A. Gemelli IRCCS, L.go A. Gemelli 8, 00168 Rome, Italy; 2 Dipartimento di Scienze Biotecnologiche di Base, Cliniche Intensivologiche e Perioperatorie, Università Cattolica del Sacro Cuore, L.go F. Vito 1, 00168 Rome, Italy

**Keywords:** vitamin D, critical care, sepsis, acute respiratory failure, acute kidney injury, acute liver dysfunction

## Abstract

Vitamin D covers roles of paramount importance in the regulation of multiple physiological pathways of the organism. The metabolism of vitamin D involves kidney–liver crosstalk and requires an adequate function of these organs, where vitamin D is progressively turned into active forms. Vitamin D deficiency has been widely reported in patients living in the community, being prevalent among the most vulnerable subjects. It has been also documented in many critically ill patients upon admission to the intensive care unit. In this context, vitamin D deficiency may represent a risk factor for the development of life-threatening clinical conditions (e.g., infection and sepsis) and worse clinical outcomes. Several researchers have investigated the impact of vitamin D supplementation showing its feasibility, safety, and effectiveness, although conflicting results have put into question its real benefit in critically ill patients. The existing studies included heterogeneous critically ill populations and used slightly different protocols of vitamin D supplementation. For these reasons, pooling up the results is difficult and not conclusive. In this narrative review, we described vitamin D physiology and the pathophysiology of vitamin D depletion with a specific focus on critically ill patients with liver dysfunction, acute kidney injury, acute respiratory failure, and sepsis.

## 1. Introduction

Vitamin D is a hormone involved in the regulation of several physiological pathways including calcium/phosphate balance, cardiovascular, anti-tumor, and immunologic homeostasis [1,2]. Its depletion may impair different metabolic functions and evidence has shown that vitamin D deficiency might be associated with multiple diseases and could worsen outcomes [1,2]. The metabolism of vitamin D requires specific enzymatic reactions in the liver and the kidney, and any alterations of these steps, caused by acute or chronic organ dysfunction, may lead to disturbed vitamin D metabolism and to a shortage of relevant vitamin D metabolites [1,2]. An increasing number of researchers have investigated the pathophysiology and the predictors of vitamin D shortage in subjects living in the community and in critically ill patients. From an epidemiological point of view, low levels of vitamin D have been frequently reported among subjects living in the community [3,4] and are also prevalent in the most vulnerable patients. Specifically, vitamin D deficiency has been reported in 70% of critically ill patients admitted to the intensive care unit (ICU) [5], which may represent a risk factor for the development of life-threatening complications [6,7,8,9]. Several researchers have investigated the impact of vitamin D supplementation in patients with vitamin D deficiency and have found that it is feasible, safe, and effective to increase the vitamin D body content [10,11], although controversial results do not support its benefit for the prognosis of critically ill patients [12,13,14,15].

In this narrative review, we describe the physiology of vitamin D and the pathophysiology of vitamin D depletion in critically ill patients. We report the epidemiology, metabolic pathways, and concurrent diseases associated with vitamin D shortage. Finally, we discuss potential options to tackle vitamin D deficiency, with a specific focus on critically ill patients.

## 2. Physiology of Vitamin D

### 2.1. Anabolic and Catabolic Pathways

Vitamin D stands for several lipophilic hormones that chemically stem from cholesterol and are characterized by a secosteroid structure [16]. Ergocalciferol (vitamin D_2_) and Cholecalciferol (vitamin D_3_) represent the most biologically powerful hormones of the vitamin D family, which also includes vitamin D_4_, vitamin D_5_, vitamin D_6_, and vitamin D_7_ [17].

**Figure 1:**
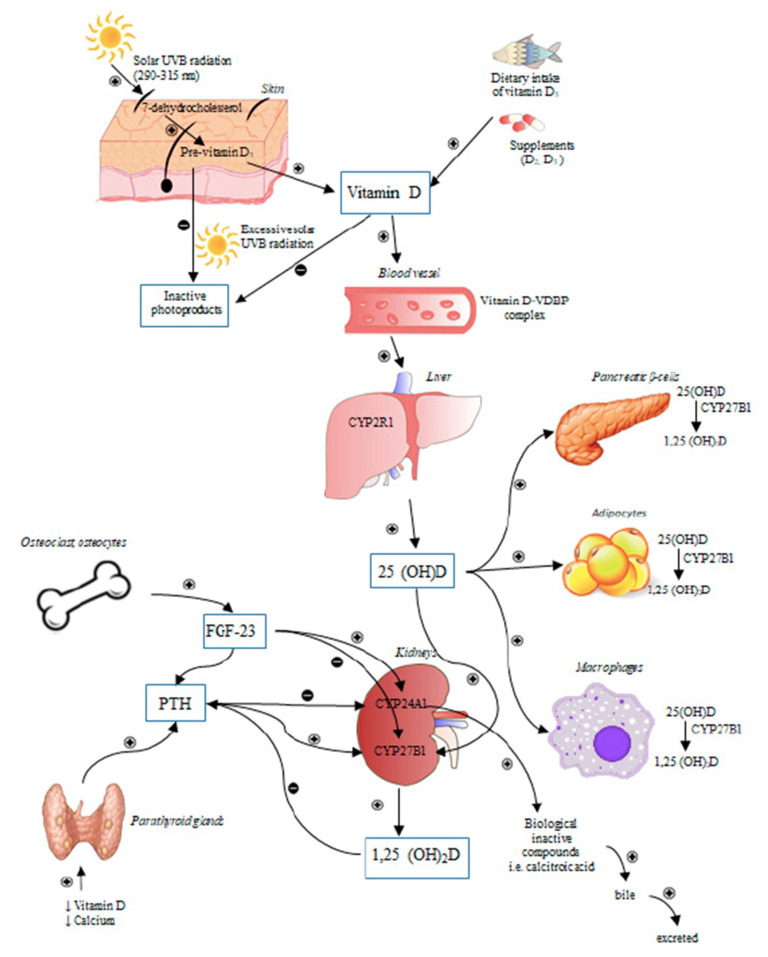
The regulation of synthesis and metabolism of vitamin D. The figure describes the anabolic and catabolic pathways of vitain D metabolism. + stimulation; − inhibition. Reprinted with permission from Szymczak-Pajor et al. [18].

The human supply of vitamin D comes mostly from vitamin D_3_, which is produced in the skin by the non-enzymatic reaction of 7-dehydrocholesterol with solar ultraviolet B radiation (wavelength between 290 and 315 nm) [18]. For this reason, vitamin D is technically a misnomer, as it differs from other vitamins because of this endogenous anabolic pathway [16]. On the other hand, a considerable amount of vitamin D is contained in a few aliments (e.g., vitamin D_2_ in plant foods, and vitamin D_3_ in fatty fish and egg), although the delivery of larger doses requires the administration of manufactured products (“fortified” food and supplements) [1]. Both vitamin D_2_ and vitamin D_3_ are stored in the adipose tissue, and circulate into the bloodstream carried by the vitamin D binding protein (LPBP), albumin (~10%) or free (<1%) [19]. Vitamin D is turned by the 25-hydroxylase of the liver (cytochrome p450, CYP2R1—endoplasmic reticulum) into the 25-hydroxyvitamin D (calcidiol), which is further converted by the 1α-hydroxylase (cytochrome p450, CYP27B1—mitochondria) of the renal tubular cells into the 1,25-dihydroxyvitamin D (calcitriol) (Figure 1). Although the 1,25-dihydroxyvitamin D is the most biologically active form of this hormone [2], 25-hydroxyvitamin D is characterized by higher blood level (1000 times) and a longer half-life (4 h vs. 2–3 weeks, respectively) [20].

The 1,25-dihydroxyvitamin production is enhanced by low levels of calcium and phosphate, via the parathormone-mediated increase in the 1α-hydroxylase activity of the kidney. On the contrary, high levels of 1,25-dihydroxyvitamin D may reduce parathormone release and foster the 24-hydroxylase activity, that catabolizes its inactivation to calcitroic acid, which is secreted into bile fluid [1] (Figure 1). In addition, 1,25-dihydroxyvitamin D production may be reduced by the fibroblastic growth factor (FGF)-23, a hormone released by osteoclasts [21].

### 2.2. Vitamin-D-Associated Metabolic Pathways

**Figure 2:**
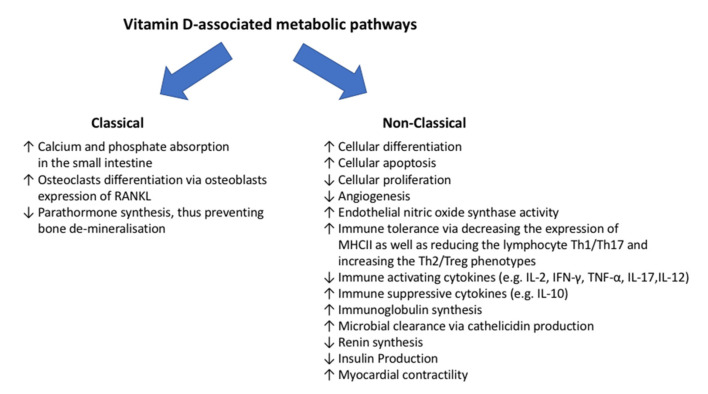
Vitamin-D-associated metabolic pathways. The figure describes both classical and non-classical metabolic pathways associated with vitamin D. IL, interleukin; MHCII, major histocompatibility complex class II; RANKL, receptor activator of nuclear factor-κB ligand; Th, T helper.

Vitamin D acts via a specific receptor, the vitamin D receptor (VDR), for which its presence in different tissues [1] allows vitamin D to exert pleiotropic activities via autocrine, paracrine, and endocrine mechanisms, that are mediated by genomic and non-genomic pathways [22,23]. Vitamin-D-associated metabolic pathways may be classified as classical and non-classical (Figure 2). Classical pathways enclose calcium and phosphate balance and, specifically, their absorption in the small intestine and storage in the bone [1]. Non-classical pathways include microbial clearance via the production of cathelicidin (LL-37) in macrophages, monocytes, and epithelial cells at the barrier level (skin, respiratory, and gastrointestinal tracts) [24]. Moreover, vitamin D induces immune tolerance [1], increases myocardial contractility [25] and nitric oxide production [26], and reduces renin [27] and insulin [28] synthesis. Furthermore, vitamin D has anti-tumor activities [29] by inducing cellular differentiation and apoptosis, as well as by reducing cell proliferation and angiogenesis [30].

## 3. Epidemiology of Vitamin D Status Alterations

### 3.1. Grading

Vitamin D status is assessed by the 25-hydroxyvitamin D blood level, whose gold standard measurement method is debated [31]. However, liquid chromatography with mass spectrometry (LC-MS) is frequently considered the reference test in the most recent clinical trials in this field [12,13]. The Institute of Medicine [32] recommend specific thresholds of 25-hydroxyvitamin D blood level (Table 1), which may help clinicians to diagnose and grade the severity of vitamin D shortage. Finally, vitamin D intoxication was identified by the 25-hydroxyvitamin D blood level above 150 ng/mL [1].

### 3.2. Epidemiology

Vitamin D deficiency is common in Europe [3] and the United States [4], where it affects 40% of subjects living in the community [20]. Although the global burden of vitamin D shortage is unknown, some reports warn about a larger prevalence in low-income countries [33]. In the ICU, vitamin D deficiency affects the 40–70% of critically ill patients and vitamin D insufficiency ranges between 20 and 40% [5].

### 3.3. Settings

Obesity [34], low dietary intake and reduced sunlight exposure due to cold seasons [35], extreme latitudes, and extensive use of sunscreens are the main risk factors for vitamin D deficiency [1]. This condition is also frequent in dark-skinned, young, and elderly subjects [3,4], and is also associated with specific physiological conditions such as pregnancy, lactation [36,37], and post-menopausal states [38]. Low vitamin D levels have been associated with several pathological conditions such as osteoporosis, muscle weakness [39], hypertension [40], atherogenic dyslipidemia [41], and metabolic syndrome [42]. Moreover, vitamin D deficiency has been reported in patients with infective diseases (e.g., tuberculosis [43], clostridium difficile [44], and COVID-19 [45]) and non-infective inflammatory states (e.g., sarcoidosis [46] or multiple sclerosis [47]). In addition, vitamin D shortage is frequent when liver dysfunction occurs [48] and may be due to enzymatic impairment and low VDBP production [5]. Low vitamin D concentrations have been constantly reported among patients with kidney dysfunction [49] and may be caused by the altered enzymatic hydroxylation of calcidiol [50,51]. Some evidence shows that vitamin D deficiency may be induced by specific drugs that induce the enzymatic catabolism of vitamin D to calcitroic acid (e.g., anticonvulsants, glucocorticoids, antiretroviral therapies, antibiotics such as rifampicin and antirejection medications) or reduce the intestinal absorption of vitamin D_2_ (e.g., cholestyramine) [1,20,52].

### 3.4. Associated Diseases

A causative link between severe vitamin D deficiency and osteoporosis/muscle weakness was ex juvantibus demonstrated by the reduction of falls and skeletal fractures in patients who received vitamin D supplementation compared with placebo [53].

Vitamin D deficiency is frequently reported in critically ill patients suffering from infection and sepsis [6,7,8,9], but the relationship between these conditions and vitamin D shortage is unknown. Moreover, the risk of developing vitamin D shortage may be increased by the limited sunlight exposure during a prolonged hospital length of stay. It is also unclear whether vitamin D depletion should be considered just a marker of clinical severity or if it represents a modifiable risk factor that may benefit from vitamin D supplementation.

## 4. Therapeutic Options of Vitamin D Supplementation

### 4.1. Routes of Vitamin D Supplementation

Vitamin D can be administered by enteral, parenteral, and intramuscular routes. Enteral formulations have been widely used in clinical trials, being characterized by their ease of administration and large vitamin D bioavailability. Specifically, some studies report a greater increase of 25-dihydroxyvitamin D blood level in patients who receive oral formulations, compared with those receiving the same dose of vitamin D by the intramuscular route [54]. The delivery of enteral formulations appears particularly convenient because it is not associated with invasive procedures (e.g., puncture or vascular access). Parenteral and intramuscular administrations of vitamin D-based compounds should be indicated for patients with malabsorption due to enteral diseases, gastrointestinal bypass surgery, and medications that reduce lipid absorption. In specific patients with low compliance to oral vitamin D supplementation, the long lasting half-life of parenteral and intramuscular compounds may be helpful [55].

### 4.2. Isoforms of Vitamin D Compounds

In clinical practice, vitamin D_2_ and vitamin D_3_ represent the most widely used isoforms of this hormone and may plausibly be considered “native vitamin D”. Although both compounds are characterized by a low stability in the presence of moisture [56], vitamin D_3_ administration is associated with a greater increase in 25-hydroxyvitamin D blood level compared with vitamin D_2_ [57]. These features are due to the chemical structure of vitamin D_3_, which favors its enzymatic activation, VDBP-mediated delivery, and VDR-specific interaction [56]. In recent years, several vitamin D analogues have been manufactured with the aim to produce compounds with enhanced pharmacokinetic and pharmacodynamic properties [58]. For example, paricalcitol does not require enzymatic activation, doxercalciferol has prolonged half-life, and maxacalcitrol specifically acts on non-classical vitamin D-associated pathways [59,60]. However, active metabolites of vitamin D such as calcitriol analogues are characterized by a narrow therapeutic index, compound instability due to rapid hydroxylation, and were demonstrated to moderately improve vitamin D body content, with no clear effects on patient clinical outcomes [58].

## 5. Vitamin D Supplementation in Critically Ill Patients

### 5.1. General Population

Vitamin D supplementation is feasible and safe in critically ill patients and has been demonstrated to be effective for improving vitamin D deficiency within few days [10,11]. However, a recent meta-analysis pooling the results of nine randomized controlled trials on 1867 critically ill patients [61] found that vitamin D supplementation had no benefit on the 28-day mortality compared with placebo (20.4% vs. 21.7%, respectively). Moreover, this intervention had no influence on the duration of mechanical ventilation, ICU, and hospital length of stay. The same meta-analysis showed that there were no specific benefits associated with the daily dose and the route of vitamin D administration [61]. In contrast, Menger et al. [62] conducted a larger systematic review and meta-analysis on 2449 critically ill patients from 16 randomized controlled trials on vitamin D supplementation, which included eight studies that were selected by Lan et al. [61] and the most recent research in this field. The authors found that vitamin D supplementation was associated with reduced overall mortality, duration of mechanical ventilation, and ICU length of stay. Furthermore, this study revealed that the parenteral administration of vitamin D was more effective than other routes for improving vitamin D deficiency. However, both systematic reviews and meta-analyses had important limitations that hampered the generalizability of results, mostly due to the wide degree of heterogeneity of the studies included. Specifically, the majority of these studies were characterized by a small sample size and applied different protocols for vitamin D supplementation. The only two large scale clinical investigations were the VITdAL-ICU [12] and the VIOLET [13] trials (Table 2), whose specific peculiarities warrant being discussed in detail.

The VITdAL-ICU trial [12] was conducted in five ICUs of an Austrian hospital and enrolled 475 critically ill patients with vitamin D deficiency, who were randomized to receive oral vitamin D supplementation (vitamin D_3_: loading dose of 540,000 IU, followed by monthly maintenance dose of 90,000 IU for 5 months) or placebo. The majority of these patients were admitted to the ICU after surgery (more than 50%), had a higher body mass index (about 27 kg/m^2^), and presented low estimated glomerular filtration rate (slightly above 60 mL/min/1.73 m^2^) at enrolment. In patients receiving vitamin D supplementation the deficiency status improved within 7 days from the inclusion and remained stable for the 28 days afterwards with respect to the placebo. Vitamin D supplementation and placebo groups were not different in terms of hospital length of stay (primary outcome); ICU length of stay; ICU mortality (22.8% vs. 26.5%, respectively); and hospital (28.3% vs. 35.5%), 28-day (21.9% vs. 28.6%, respectively), and 6-month mortalities (35% vs. 42.9%, respectively) (secondary outcomes). A subgroup analysis of patients with severe vitamin D deficiency (25-hydroxyvitamin D levels ≤ 12 ng/mL) showed that vitamin D supplementation reduced the risk of hospital mortality compared with placebo (28.6% vs. 46.1%, respectively). In light of this finding, the VIOLET trial [13] was designed and conducted in 44 hospitals in the United States. This study enrolled 1358 severe patients with vitamin D deficiency, who were deemed to be managed in the ICU and were randomized to receive enteral vitamin D supplementation (Vitamin D_3_: loading dose of 540,000 IU, administered even before ICU admission and not followed by a maintenance dose) or placebo. The majority of these patients were admitted to the ICU for medical diseases (more than 80%), had a high body mass index (about 30 kg/m^2^), and presented a low estimated glomerular filtration rate (about 60 mL/min/1.73 m^2^) at the enrolment. About 20% of these patients were Afro-American. Although vitamin D status improved in patients who received vitamin D supplementation compared with placebo, this intervention had no impact on mortality rate at 90 days (23.5% vs. 20.6%, respectively) (primary outcome), 28 days (17.3% vs. 13.1%, respectively), and at hospital discharge (17.1% vs. 13.4%) (secondary outcomes). In both trials [12,13], vitamin D supplementation was feasible, safe, and not associated with significant adverse events compared with placebo. However, the VITdAL-ICU [12] and the VIOLET [13] trials enrolled patients with different characteristics according to the type of ICU admission (surgical vs. medical), demographics (white vs. black), and exposure to vitamin D supplementation (maintenance dose vs. no maintenance dose), which have limited the pooling of the results and justify the significant degree of heterogeneity among these studies. Many patients included in both trials were overweight and had impaired renal function, thus implying that larger vitamin doses should have been given when participants were at high risk of vitamin D deficiency, in order to reliably test the efficacy on clinical outcomes. Finally, no studies have ever assessed whether the underlying cause of vitamin D shortage was due to impairment of the enzymes involved in the anabolic pathways of this hormone [63]. In this condition, unspecific supplementation strategies may fail to improve vitamin D levels and should be oriented by the assessment of the vitamin D metabolite distribution provided by LC-MS.

### 5.2. Acute Liver Dysfunction

The relationship between vitamin D status and acute liver dysfunction (ALF) has been poorly investigated. Although it is physiologically plausible that ALF may lower vitamin D status due to impaired 25-hydroxylase activity and VDBP production, no strong clinical evidence exists on the effect of vitamin D supplementation in this population. Recently, a retrospective study [64] on 528 patients who underwent liver transplantation showed that 55% of them presented vitamin D deficiency and this condition was correlated with the Model for End-Stage Liver Disease-Na (MELD-Na) score [65]. Moreover, post-operative vitamin D deficiency was a predictor of mortality and the post-transplant vitamin D supplementation was associated with a low risk of acute cellular rejection of the liver [65]. In this setting, Martucci et al. [66] conducted a prospective observational study in orthotopic liver transplantation recipients and observed that the majority of them were vitamin D deficient before surgery. In this study, low vitamin D levels before surgery were correlated with severe liver dysfunction at baseline, were associated with the development of infection within 28 days afterwards, and, when persistent at this timepoint, predicted incomplete graft recovery [66]. Accordingly, further investigations are strongly advocated to clarify the role of vitamin D shortage and vitamin D supplementation in this clinical scenario.

### 5.3. Acute Kidney Injury

Acute kidney injury (AKI) may lead to vitamin D deficiency, mostly due to the negative feedback exerted by phosphate and FGF-23 accumulation on 1α-hydroxylase [67]. On the contrary, vitamin D deficiency may lower renin activity and cause alteration of the renal microcirculation. In this setting, the VID-AKI study [68] is ongoing and will provide observational evidence on the distribution of vitamin D status in critically ill patients with and without AKI. Moreover, the role of vitamin D in AKI is unclear and no studies have ever assessed whether vitamin D supplementation may improve the outcome.

### 5.4. Acute Respiratory Failure

Several studies have reported that vitamin D deficient status is associated with worse clinical outcomes in patients with acute respiratory failure [69] caused by COVID-19 [45], sepsis, and trauma [70]. Recently, Murai et al. [15] investigated the immunomodulatory effect of vitamin D supplementation in 240 hospitalized patients with moderate to severe COVID-19 disease, who were randomized to receive either oral vitamin D_3_ (single dose: 200,000 IU) or placebo. The majority of these patients were not critically ill, were hypoxemic, and required supplemental oxygen at baseline (212 patients, 89.5%), whereas the distribution and severity of ARDS was not reported in the manuscript. Although vitamin D blood levels improved in patients who received vitamin D supplementation compared with placebo, there was no clinical benefit associated with this intervention in terms of hospital length of stay, admission to the ICU, need for mechanical ventilation, and hospital mortality. Pre-specified and post hoc exploratory analyses of this trial found that vitamin D supplementation had no influence on the bloodstream proinflammatory and immunosuppressive mediator concentration in comparison with the placebo [71]. In this trial, almost 50% of patients were at risk of vitamin D shortage because of black ethnicity and obesity, which corresponded to a prevalence of vitamin D insufficiency of 50%.

Finally, it is unknown whether vitamin D deficiency and acute respiratory failure have a causal link and whether the effect of vitamin D administration to provide immunomodulation may exert some benefit. Accordingly, this therapy in patients with acute respiratory failure should not be recommended with the aim of improving clinical outcomes.

### 5.5. Sepsis

Sepsis is a clinical syndrome and initiates from a dysregulated host response to infection, which alters the interplay between different systems of the host and leads to multi-organ dysfunction [72]. Specifically, a balanced immune system response to pathogens is of pivotal importance to protect the host from microbiological threats, and its alteration may be associated with worse clinical outcomes [73]. In this context, vitamin D deficiency was plausibly associated with immune dysfunction and represented a risk factor for the development of infection [6,7,8,9]. Moromizato et al. [74] observed that the 25-hydroxyvitamin D blood levels ≤ 15 ng/mL before hospital admission was predictive for the risk of sepsis and 90-day mortality in 3386 patients. Moreover, De Pascale et al. [9] observed that extremely low 25-hydroxyvitamin D blood levels (<7 ng/mL) upon admission to the ICU were independently associated with sepsis-related mortality in 107 critically ill septic patients. Specifically, patients with extremely low 25-hydroxyvitamin D blood levels were characterized by lower microbiological eradication, longer duration of mechanical ventilation, and vasopressor support compared with those who had vitamin D blood levels ≥ 7 ng/mL [9].

A systematic review and meta-analysis showed that vitamin D deficiency in critically ill patients admitted to the ICU with sepsis was independently associated with an increased risk of mortality [75]. In light of this, 67 critically ill patients with sepsis were randomly assigned to receive 2 μg of intravenous calcitriol or placebo [14], with the aim of testing the hypothesis that vitamin D supplementation may improve cathelicidin blood levels within 24 h (primary outcome), increase the 1,25-dihydroxyvitamin D blood level within 6 h, influence the cytokine mRNA expression and cytokine levels in the bloodstream within 24 h, and reduce urinary markers of kidney injury within 48 h (secondary outcomes). Although vitamin D blood levels increased in patients who received vitamin D supplementation, it was only associated with an increase in cathelicidin and IL-10 mRNA expression within 24 h compared with the placebo. Vitamin D supplementation was not associated with 28-day ICU and hospital mortality compared with the placebo; however, this trial was not powered to assess the impact of vitamin D supplementation on patient outcome. Furthermore, the VITdAL-ICU [12] and VIOLET [13] trials included critically ill patients with a low prevalence of sepsis at the enrollment (7.7% and 33.3%), for whom the subgroup analyses did not allow for drawing a firm conclusion on the efficacy of vitamin D supplementation in this clinical condition. Accordingly, future trials are warranted in this field and should include the use of specific biomarkers of immune dysfunction, besides vitamin D blood levels, in order to improve the selection of septic patients possibly resolving the heterogeneity among the different trials [73].

## 6. Conclusions

Vitamin D deficiency is frequent in the community and is prevalent in critically ill patients. Although several studies have observed an association between low vitamin D blood level and life-threatening diseases leading to ICU admission, no causative link has ever been proven. Moreover, randomized controlled trials on vitamin D supplementation in various populations of critically ill patients failed to demonstrate the efficacy of this intervention to improve clinical outcomes. However, the paucity of large-scale clinical investigations and the high degree of patient heterogeneity among different studies render problematic the pooling of the results and drawing solid conclusions on this topic. For this reason, the role of vitamin D in the pathophysiology of liver dysfunction, acute kidney injury, acute respiratory failure, and sepsis remains unclear and high quality clinical trials are thus warranted to clarify whether vitamin D supplementation may help to improve the outcome of critically ill patients.

## Figures and Tables

**Table 1:** Grading of vitamin D shortage.

Grading	Institute of Medicine [32]
Vitamin D	
Deficiency	25(OH)D blood level ≤ 20 ng/mL
Insufficiency	25(OH)D blood level 21–29 ng/mL
Sufficiency	25(OH)D blood level ≥ 30 ng/mL

**Table 2:** Large-scale randomized clinical trials on vitamin D supplementation in critically ill patients.

Authors, Year of Publication	Study Sites	Study Duration	Number of Patients	Inclusion Criteria	Intervention	Primary Outcome	Patients Characteristics	Main Result
Amrein et al. (the VITdAL-ICU trial), 2014 [12]	Single centre, Austria	2012–2015	475	Adult white critically ill patients, expected length of ICU stay ≥48 h and with 25-hydroxyvitamin D blood level of ≤20 ng/mL	Enteral vitamin D3 protocol administration: 540,000 IUs followed by monthly 90,000 IU for 5 months vs. Placebo	Length of hospital stay	Surgical patients were prevalent Mean body mass index about 27 kg/m^2^ Mean eGRF slightly above 60 mL/min/1.73 m^2^	No difference for the primary outcome
Ginde et al. (the VIOLET trial), 2019 [13]	44 centres, USA	2017–2018	1078	Adult patients with with >1 risk factors for death or lung injury, deemed to be managed in the ICU and with 25-hydroxyvitamin D blood level ≤20 ng/mL	Enteral vitamin D3 protocol administration: 540,000 Ius vs. Placebo	90-day mortality rate	Medical patients were prevalent Black patients about 20% Mean body mass index about 30 kg/m^2^ Mean eGRF slightly about 60 mL/min/1.73 m^2^	No difference for the primary outcome

eGFR, estrimated glomerular filtration rate; ICU, intensive care unit.

## Data Availability

Not applicable.

## References

[1] Holick M. (2007). Vitamin D deficiency. N. Engl. J. Med..

[2] Rosen C. (2011). Clinical practice. Vitamin d insufficiency. N. Engl. J. Med..

[3] Cashman K., Dowling K., Škrabáková Z., Gonzalez-Gross M., Valtueña J., Henauw S.D., Moreno L., Damsgaard C., Michaelsen K., Mølgaard C. (2016). Vitamin D deficiency in europe: Pandemic?. Am. J. Clin. Nutr..

[4] Herrick K., Storandt R., Afful J., Pfeiffer C., Schleicher R., Gahche J., Potischman N. (2019). Vitamin D status in the United States, 2011–2014. Am. J. Clin. Nutr..

[5] Amrein K., Papinutti A., Mathew E., Vila G., Parekh D. (2018). Vitamin D and critical illness: What endocrinology can learn from intensive care and vice versa. Endocr. Connect.

[6] de Haan K., Groeneveld A., de Geus H., Egal M., Struijs A. (2014). Vitamin D deficiency as a risk factor for infection, sepsis and mortality in the critically ill: Systematic review and meta-analysis. Crit. Care.

[7] McNally J., Nama N., O’Hearn K., Sampson M., Amrein K., Iliriani K., McIntyre L., Fergusson D., Menon K. (2017). Vitamin D deficiency in critically ill children: A systematic review and meta-analysis. Crit. Care.

[8] Parekh D., Patel J., Scott A., Lax S., Dancer R., D’Souza V., Greenwood H., Fraser W., Gao F., Sapey E. (2017). Vitamin D deficiency in human and murine sepsis. Crit. Care Med..

[9] De Pascale G., Vallecoccia M., Schiattarella A., Di Gravio V., Cutuli S., Bello G., Montini L., Pennisi M., Spanu T., Zuppi C. (2016). Clinical and microbiological outcome in septic patients with extremely low 25-hydroxyvitamin D levels at initiation of critical care. Clin. Microbiol. Infect..

[10] Quraishi S., De Pascale G., Needleman J., Nakazawa H., Kaneki M., Bajwa E., Jr C.C., Bhan I. (2015). Effect of cholecalciferol supplementation on vitamin D status and cathelicidin levels in sepsis: A randomized, placebo-controlled trial. Crit. Care Med..

[11] Amrein K., Sourij H., Wagner G., Holl A., Pieber T., Smolle K., Stojakovic T., Schnedl C., Dobnig H. (2011). Short-term effects of high-dose oral vitamin D3 in critically ill vitamin d deficient patients: A randomized, double-blind, placebo-controlled pilot study. Crit. Care.

[12] Amrein K., Schnedl C., Holl A., Riedl R., Christopher K., Pachler C., Purkart T.U., Waltensdorfer A., Münch A., Warnkross H. (2014). Effect of high-dose vitamin D3 on hospital length of stay in critically ill patients with vitamin d deficiency: The vitdal-icu randomized clinical trial. JAMA.

[13] Ginde A.A., Brower R., Caterino J., Finck L., Banner-Goodspeed V., Grissom C., Hayden D., Hough C., Hyzy R., National Heart, Lung, and Blood Institute PETAL Clinical Trials Network (2019). Early high-dose vitamin D 3 for critically ill, vitamin D-deficient patients. N. Engl. J. Med..

[14] Leaf D., Raed A., Donnino M., Ginde A., Waikar S. (2014). Randomized controlled trial of calcitriol in severe sepsis. Am. J. Respir. Crit. Care Med..

[15] Murai I., Fernandes A., Sales L., Pinto A., Goessler K., Duran C., Silva C., Franco A., Macedo M., Dalmolin H. (2021). Effect of a single high dose of vitamin D3 on hospital length of stay in patients with moderate to severe COVID-19: A randomized clinical trial. JAMA.

[16] Demer L., Hsu J., Tintut Y. (2018). Steroid hormone vitamin D: Implications for cardiovascular disease. Circ. Res..

[17] Komba S., Kotake-Nara E., Tsuzuki W. (2019). Simultaneous synthesis of vitamins D 2, D 4, D 5, D 6, and D 7 from commercially available phytosterol, β-sitosterol, and identification of each vitamin d by hsqc nmr. Metabolites.

[18] Szymczak-Pajor I., Śliwińska A. (2019). Analysis of association between vitamin D deficiency and insulin resistance. Nutrients.

[19] De Pascale G., Quraishi S. (2014). Vitamin D status in critically ill patients: The evidence is now bioavailable!. Crit. Care.

[20] Holick M.F., Binkley N.C., Bischoff-Ferrari H.A., Gordon C.M., Hanley D.A., Heaney R.P., Murad M.H., Weaver C.M., Endocrine S. (2011). Evaluation, treatment, and prevention of vitamin D deficiency: An endocrine society clinical practice guideline. J. Clin. Endocrinol Metab..

[21] Berndt T., Kumar R. (2007). Phosphatonins and the regulation of phosphate homeostasis. Annu. Rev. Physiol..

[22] Prentice A., Goldberg G., Schoenmakers I. (2008). Vitamin D across the lifecycle: Physiology and biomarkers. Am. J. Clin. Nutr..

[23] Norman A. (2008). From vitamin D to hormone d: Fundamentals of the vitamin d endocrine system essential for good health. Am. J. Clin. Nutr..

[24] Quraishi S., Camargo C.J. (2012). Vitamin D in acute stress and critical illness. Curr. Opin. Clin. Nutr. Metab. Care.

[25] Zittermann A. (2006). Vitamin D and disease prevention with special reference to cardiovascular disease. Prog. Biophys. Mol. Biol..

[26] Molinari C., Uberti F., Grossini E., Vacca G., Carda S., Invernizzi M., Cisari C. (2011). 1α,25-dihydroxycholecalciferol induces nitric oxide production in cultured endothelial cells. Cell. Physiol. Biochem..

[27] Li Y. (2003). Vitamin D regulation of the renin-angiotensin system. J. Cell. Biochem..

[28] Chiu K., Chu A., Go V., Saad M. (2004). Hypovitaminosis D is associated with insulin resistance and beta cell dysfunction. Am. J. Clin. Nutr..

[29] Chandler P., Chen W., Ajala O., Hazra A., Cook N., Bubes V., Lee I., Giovannucci E., Willett W., Buring J. (2020). Effect of vitamin D3 supplements on development of advanced cancer: A secondary analysis of the vital randomized clinical trial. JAMA Netw Open.

[30] Umar M., Sastry K., Chouchane A. (2018). Role of vitamin D beyond the skeletal function: A review of the molecular and clinical studies. Int. J. Mol. Sci..

[31] Makris K., Sempos C., Cavalier E. (2020). The measurement of vitamin D metabolites: Part i-metabolism of vitamin D and the measurement of 25-hydroxyvitamin D. Hormones.

[32] Institute of Medicine (US) (2011). Committee to Review Dietary Reference Intakes for Vitamin D and Calcium. Dietary Reference Intakes for Calcium and Vitamin D.

[33] El Hajj Fuleihan G., Nabulsi M., Choucair M., Salamoun M., Shahine C.H., Kizirian A., Tannous R. (2001). Hypovitaminosis D in healthy schoolchildren. Pediatrics.

[34] Migliaccio S., Di Nisio A., Mele C., Scappaticcio L., Savastano S., Colao A., Obesity Programs of nutrition, Education, Research and Assessment (OPERA) Group (2019). Obesity and hypovitaminosis D: Causality or casualty?. Int. J. Obes. Suppl..

[35] Amrein K., Zajic P., Schnedl C., Waltensdorfer A., Fruhwald S., Holl A., Purkart T., Wünsch G., Valentin T., Grisold A. (2014). Vitamin D status and its association with season, hospital and sepsis mortality in critical illness. Crit. Care.

[36] Lee J., Smith J., Philipp B., Chen T., Mathieu J., Holick M. (2007). Vitamin D deficiency in a healthy group of mothers and newborn infants. Clin. Pediatr..

[37] Wagner C., Taylor S., Dawodu A., Johnson D., Hollis B. (2012). Vitamin D and its role during pregnancy in attaining optimal health of mother and fetus. Nutrients.

[38] Lips P., Hosking D., Lippuner K., Norquist J., Wehren L., Maalouf G., Ragi-Eis S., Chandler J. (2006). The prevalence of vitamin D inadequacy amongst women with osteoporosis: An international epidemiological investigation. J. Intern. Med..

[39] Lips P., Schoor N.V. (2011). The effect of vitamin D on bone and osteoporosis. Best Pract. Res. Clin. Endocrinol. Metab..

[40] Alagacone S., Verga E., Verdolini R., Saifullah S. (2020). The association between vitamin D deficiency and the risk of resistant hypertension. Clin. Exp. Hypertens.

[41] Han Y., Hsu S., Su T. (2021). Association between vitamin D deficiency and high serum levels of small dense ldl in middle-aged adults. Biomedicines.

[42] Kim Y., Hwang J., Song M. (2018). The association between vitamin D deficiency and metabolic syndrome in korean adolescents. J. Pediatr. Nurs..

[43] Wang C., Hu Y., Wang Y., Chen C., Lai C., Huang K. (2019). Association between vitamin D and latent tuberculosis infection in the United States: Nhanes, 2011-2012. Infect. Drug Resist..

[44] Furuya-Kanamori L., Wangdi K., Yakob L., McKenzie S., Doi S., Clark J., Paterson D., Riley T., Clements A. (2017). 25-hydroxyvitamin D concentrations and clostridium difficile infection: A meta-analysis. JPEN J. Parenter. Enteral Nutr..

[45] Oscanoa T., Amado J., Vidal X., Laird E., Ghashut R., Romero-Ortuno R. (2021). The relationship between the severity and mortality of SARS-CoV-2 infection and 25-hydroxyvitamin D concentration—A metaanalysis. Adv. Respir. Med..

[46] Kiani A., Abedini A., Adcock I., Mirenayat M., Taghavi K., Mortaz E., Kazempour-Dizaji M. (2018). Association between vitamin D deficiencies in sarcoidosis with disease activity, course of disease and stages of lung involvements. J. Med. Biochem..

[47] Sintzel M., Rametta M., Reder A. (2018). Vitamin D and multiple sclerosis: A comprehensive review. Neurol. Ther..

[48] Männistö V., Jääskeläinen T., Färkkilä M., Jula A., Männistö S., Lundqvist A., Zeller T., Blankenberg S., Salomaa V., Perola M. (2021). Low serum vitamin D level associated with incident advanced liver disease in the general population—A prospective study. Scand J. Gastroenterol..

[49] Jean G., Souberbielle J., Chazot C. (2017). Vitamin D in chronic kidney disease and dialysis patients. Nutrients.

[50] Leaf D., Wolf M., Waikar S., Chase H., Christov M., Cremers S., Stern L. (2012). Fgf-23 levels in patients with AKI and risk of adverse outcomes. Clin. J. Am. Soc. Nephrol..

[51] Bacchetta J., Sea J., Chun R., Lisse T., Wesseling-Perry K., Gales B., Adams J., Salusky I., Hewison M. (2013). Fibroblast growth factor 23 inhibits extrarenal synthesis of 1,25-dihydroxyvitamin D in human monocytes. J. Bone Miner. Res..

[52] Holick M. (2006). High prevalence of vitamin D inadequacy and implications for health. Mayo Clin. Proc..

[53] Thanapluetiwong S., Chewcharat A., Takkavatakarn K., Praditpornsilpa K., Eiam-Ong S., Susantitaphong P. (2020). Vitamin D supplement on prevention of fall and fracture: A meta-analysis of randomized controlled trials. Medicine.

[54] Romagnoli E., Mascia M., Cipriani C., Fassino V., Mazzei F., D’Erasmo E., Carnevale V., Scillitani A., Minisola S. (2008). Short and long-term variations in serum calciotropic hormones after a single very large dose of ergocalciferol (vitamin D2) or cholecalciferol (vitamin D3) in the elderly. J. Clin. Endocrinol. Metab..

[55] Vora L., VG S., Vavia P. (2017). Zero order controlled release delivery of cholecalciferol from injectable biodegradable microsphere: In-vitro characterization and in-vivo pharmacokinetic studies. Eur. J. Pharm. Sci..

[56] Gupta R., Behera C., Paudwal G., Rawat N., Baldi A., Gupta P. (2018). Recent advances in formulation strategies for efficient delivery of vitamin D. AAPS PharmSciTech.

[57] Heaney R., Recker R., Grote J., Horst R., Armas L. (2011). Vitamin D(3) is more potent than vitamin D(2) in humans. J. Clin. Endocrinol. Metab..

[58] Bilezikian J., Formenti A., Adler R., Binkley N., Bouillon R., Lazaretti-Castro M., Marcocci C., Napoli N., Rizzoli R., Giustina A. (2021). Vitamin D: Dosing, levels, form, and route of administration: Does one approach fit all?. Rev. Endocr. Metab. Disord..

[59] Chen J., Tang Z., Slominski A., Li W., Żmijewski M., Liu Y., Chen J. (2020). Vitamin D and its analogs as anticancer and anti-inflammatory agents. Eur. J. Med. Chem..

[60] Cunningham J., Zehnder D. (2011). New vitamin D analogs and changing therapeutic paradigms. Kidney Int..

[61] Lan S., Lai C., Chang S., Lu L., Hung S., Lin W. (2020). Vitamin D supplementation and the outcomes of critically ill adult patients: A systematic review and meta-analysis of randomized controlled trials. Sci. Rep..

[62] Menger J., Lee Z., Notz Q., Wallqvist J., Hasan M.S., Elke G., Dworschak M., Meybohm P., Heyland D., Stoppe C. (2022). Administration of vitamin D and its metabolites in critically ill adult patients: An updated systematic review with meta-analysis of randomized controlled trials. Crit. Care.

[63] Saponaro F., Saba A., Zucchi R. (2020). An update on vitamin D metabolism. Int. J. Mol. Sci..

[64] Doi J., Moro A., Fujiki M., Eghtesad B., Quintini C., Menon K.N., Hashimoto K., Sasaki K. (2020). Nutrition support in liver transplantation and postoperative recovery: The effects of vitamin D level and vitamin D supplementation in liver transplantation. Nutrients.

[65] Biggins S., Kim W., Terrault N., Saab S., Balan V., Schiano T., Benson J., Therneau T., Kremers W., Wiesner R. (2006). Evidence-based incorporation of serum sodium concentration into MELD. Gastroenterology.

[66] Martucci G., Volpes R., Panarello G., Tuzzolino F., Di Carlo D., Ricotta C., Gruttadauria S., Conaldi P., Luca A., Amrein K. (2021). Vitamin D levels in liver transplantation recipients and early postoperative outcomes: Prospective observational dliverx study. Clin. Nutr..

[67] Christov M., Waikar S., Pereira R., Havasi A., Leaf D., Goltzman D., Pajevic P., Wolf M., Jüppner H. (2013). Plasma fgf23 levels increase rapidly after acute kidney injury. Kidney Int..

[68] Cameron L., Lei K., Smith S., Doyle N., Doyle J., Flynn K., Purchase N., Smith J., Chan K., Kamara F. (2017). Vitamin D levels in critically ill patients with acute kidney injury: A protocol for a prospective cohort study (vid-aki). BMJ Open.

[69] Dancer R., Parekh D., Lax S., D’Souza V., Zheng S., Bassford C., Park D., Bartis D., Mahida R., Turner A. (2015). Vitamin D deficiency contributes directly to the acute respiratory distress syndrome (ards). Thorax.

[70] Barnett N., Zhao Z., Koyama T., Janz D., Wang C., May A., Bernard G., Ware L. (2014). Vitamin D deficiency and risk of acute lung injury in severe sepsis and severe trauma: A case-control study. Ann. Intensive Care.

[71] Fernandes A., Murai I., Reis B., Sales L., Santos M., Pinto A., Goessler K., Duran C., Silva C., Franco A. (2022). Effect of a single high dose of vitamin D3 on cytokines, chemokines, and growth factor in patients with moderate to severe COVID-19. Am. J. Clin. Nutr..

[72] Singer M., Deutschman C., Seymour C., Shankar-Hari M., Annane D., Bauer M., Bellomo R., Bernard G., Chiche J., Coopersmith C. (2016). The third international consensus definitions for sepsis and septic shock (sepsis-3). JAMA.

[73] Cutuli S., Carelli S., Grieco D., De Pascale G. (2021). Immune modulation in critically ill septic patients. Medicina.

[74] Moromizato T., Litonjua A., Braun A., Gibbons F., Giovannucci E., Christopher K. (2014). Association of low serum 25-hydroxyvitamin D levels and sepsis in the critically ill. Crit. Care Med..

[75] Li Y., Ding S. (2020). Serum 25-hydroxyvitamin D and the risk of mortality in adult patients with sepsis: A meta-analysis. BMC Infect. Dis..

